# Traditional Medicine in Oman: Its Role in Ophthalmology

**DOI:** 10.4103/0974-9233.53869

**Published:** 2009

**Authors:** Radha Shenoy, Alexander Bialasiewicz, Rajiv Khandekar, Badar Al Barwani, Habiba Al Belushi

**Affiliations:** From the Department of Ophthalmology, Armed Forces Hospital, Sultanate of Oman; 1 From the Department of Ophthalmology, Al Ahli Hospital, Doha, Qatar; 2 From the Institute of Health Sciences, Ministry of Health, Sultanate of Oman; 3 From the Department of Ophthalmology, Sultan Qaboos University, Sultanate of Oman

**Keywords:** Herbal medicine, ocular injury, traditional medicine, wasam

## Abstract

**Aim:**

To present three patients with ocular disease who developed a range of complications following use of traditional medications.

**Settings and Design:**

Case series

**Methods:**

Three patients who were examined in the Ophthalmic department of a tertiary care teaching hospital in the Sultanate of Oman between 2003 and 2004, seeking care following use of traditional medicines and or healing practices for various ophthalmic problems described below.

**Results:**

The first patient was a computer professional with a chalazion; the patient used a plant extract from ‘*Calotropis procera*’ as a part of the treatment. He developed corneal edema with decrease in vision in his left eye following application of the plant extract. Treatment with topical steroids and antibiotics resulted in a complete clinical and visual recovery. The second patient developed a fungal corneal ulcer (dermatophyte - *Trichophyton mentagrophyte*) after sustaining injury with an animal tail to the right eye and used honey for pain relief prior to presentation. She responded poorly to anti-fungal treatment, underwent a penetrating keratoplasty with recurrence of infection in the graft that resulted in a vascularized corneal scar. The third patient was a five-year-old child who was treated with ‘wasam’ on the occiput for intraocular inflammation following bilateral uncomplicated cataract extraction. Following this treatment the topical steroid was discontinued. The ‘Wasam’ treatment indirectly resulted in exacerbation of the intraocular inflammation and secondary glaucoma and poor vision as well as ‘Wasam ulcers’ on the occiput. Despite treatment of the intraocular inflammation, the visual outcome was poor.

**Conclusion:**

Traditional medicine in Oman is sought by many for variable reasons. Lack of evidence-based scientific data on its safety or efficacy does not deter the Omanis from flocking the traditional healers. However, when applied in the treatment of ocular diseases, traditional medicine and healing practices seem to cause more harm than benefit for the patient.

## INTRODUCTION

Globalization and cross movement of people across countries have lead to adoption of traditional health practices, once indigenous to a sect of population or country, by other populations outside its indigenous culture.^1^ It has been noted that approximately about 80% of population from the developing as well as the developed countries use such traditional services either for diagnosis, treatment, prevention of disease and/ or maintenance of good health.^1^ If utilized properly, traditional medicine and health practices can be a source of income as has already been described in countries like India and China, where traditional health practices have evolved and developed over the years and are accepted the world over as established modes of alternative therapies.^1^–^7^ Other countries like Nepal have integrated traditional medicine in Public Health Centers and used trained traditional healers for health promotion to the community. However, inappropriate use can be harmful and have deleterious effects on health.^2^

We report three cases in which damaging ocular effects following usage of alternative or traditional medicine for treatment of eye disorders was observed.

## MATERIALS AND METHODS

Three patients who were examined in the Ophthalmic department of a tertiary care teaching hospital in the Sultanate of Oman, between 2003 and 2004, seeking care, following use of traditional medicines and or healing practices for various ophthalmic problems are described below.

## RESULTS (CASE REPORTS)

### Case 1

A 22-year-old Omani computer professional presented with painless loss of vision in his left eye following the single application of the juice from the plant ‘*Calotropis. procera*’, also known to the local population as ‘shakr/ushaar’. Extract from various parts of the plant is used for the treatment of arthritis, arthralgia and for other diseases. He used this on advice of a friend, to treat the chalazion involving the left lower lid.

**Figure 1:**
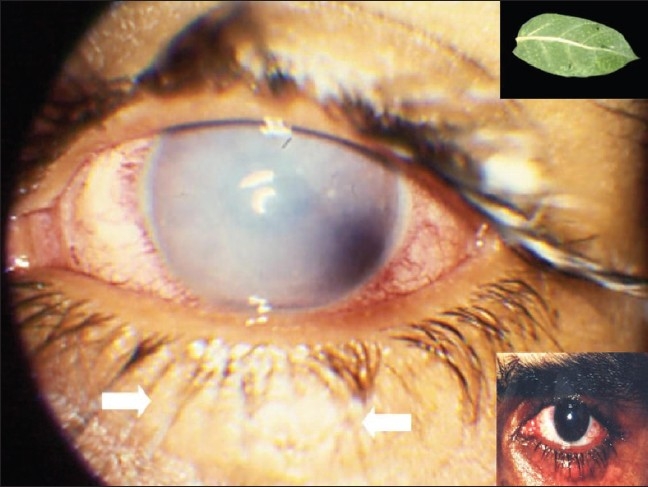
Photograph showing hazy cornea in the eye treated with the leaf of *Calotropis procera* and the same eye with clear cornea and chalazion lower lid after treatment (upper and lower inset, respectively)

On examination the best corrected visual acuity in the right eye was 1.0(-0.25 D sph/-0.50 Dcyl × 30°) and left eye was perception of light (LP). Slit lamp examination of left eye showed conjunctival pallor. The cornea was edematous and hazy. There was no fluorescein staining of the cornea. Intraocular pressure was 10 mmHg. The corneal edema prevented detailed examination of deeper structures of eye. The patient was treated with frequent topical steroids (1% Prednisolone acetate (Predforte^®^ Allergan Inc, Irvine, CA) every hour and with an antibiotic (Ofloxacin 0.3%/Exocin^®^ Allergan Inc, Irvine, CA) four times daily and a cycloplegic (Tropicamide 1% w/v (Mydriacyl^®^ Alcon Inc., Ft Worth TX) three times daily. Ten days later the corneal cleared and the visual acuity was 1.0 in the left eye. There chalazion on the left lower lid remained unchanged (Figure 1).

### Case 2

A 35-year-old lady, who worked in the royal stable, presented with redness and decreased vision in her right eye of 15 days duration. She was initially treated at a local health center for a corneal abrasion in her right eye, after being hit by the tail of a horse while cleaning the stable. She applied honey three times everyday to her painful right eye as advised by a local traditional healer. A week later, the pain in her eye decreased, but the vision in the right eye deteriorated and redness increased.

At the time of presentation, she had no pain or photophobia. The best corrected vision in the right eye was counting fingers at 2 m, and left eye was 0.5 with +2.0 D sph. The right eye showed ciliary congestion. A central corneal ulcer measuring (2 × 3 mm) in size with a hypopyon was noted. Culture of the scrapings from the ulcer grew dermatophytes on Sabouraud agar, which was identified as *T. mentagrophytes*. There was no evidence of superficial mycoses affecting skin, nails or any other area of her body.

**Figure 2:**
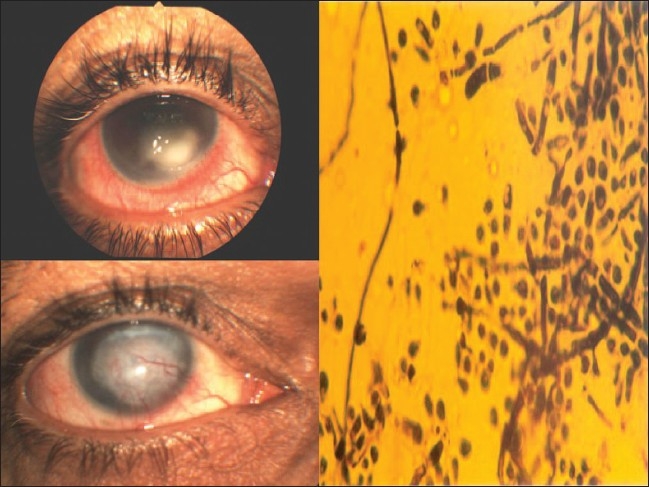
Photograph showing the hypopyon corneal ulcer and vascularized scar on the right. *Trichophyton mentagrophytes* in Sabouraud's media on the left

The ulcer was treated with topical antifungal drops (Fluconazole 0.3%; Zocon^®^ FDA Ltd) every hour, and cycloplegic (Cyclopentolate hydrochloride 1%; Mydrilate^®^ Intrapharm) three times daily. Ten days following treatment, the patient underwent therapeutic keratoplasty for a non-healing corneal ulcer with impending perforation. The graft was clear and the eye quiet with a visual acuity of 0.2 on the first postoperative day. The topical medications were continued post-keratoplasty. However, the primary infection recurred involving the graft by the fourth postoperative day, progressing relentlessly to involve the entire graft by the 15th day resulting in a vascularized central scar by 2 months (Figure 2). Posterior segment of the eye and the vitreous cavity appeared normal on B scan ultrasonography. Intra ocular pressure was 10 mmHg. The final visual acuity at the end of 2 months was appreciation of hand movements in the right eye.

### Case 3

A five-year-old female, child of a medical assistant working in a tertiary care teaching hospital in the Sultanate of Oman, underwent bilateral routine uneventful cataract extraction and posterior chamber intraocular lens implantation for congenital cataracts. She presented with painful red eyes two weeks post-surgery. The parents had discontinued the postoperative topical steroid (Prednisolone acetate 1%; Predforte^®^ Allergan Inc, Irvine CA) administered four times daily and antibiotic drops (Ofloxacin 0.3% w/v; Exocin^®^ Allergan, Irvine CA) administered four times daily and was instead treated with ‘Wasam’ treatment. At the time of presentation, the vision in the right eye was hand motions and the left eye was 1/50. Both eyes showed conjunctival congestion, corneal edema, retrocorneal membrane formation, posterior synechiae and secondary glaucoma (left eye 25 mmHg, right eye 40 mmHg). B scan ultrasound showed normal posterior segments in both the eyes.

There were two circular ulcerated areas on her occiput of about 4 cm diameter.

**Figure 3:**
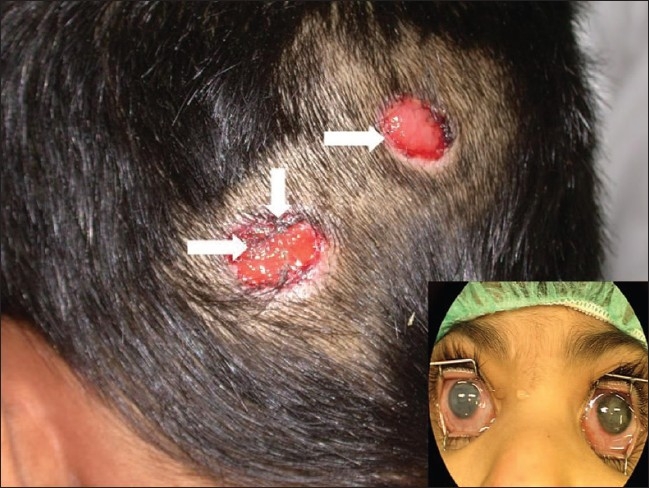
Photograph showing the wasam marks on the occiput. Bilateral inflamed eyes (inset)

The child was hospitalized for over one month to control the intraocular inflammation, secondary glaucoma and to ensure that the ulcers on the occipital region healed (Figure 3). Six months later, the secondary glaucoma had resolved, but vision remained unchanged (right eye hand motions and left eye, 1/50). The child had scars of ‘wasam’ in the occipital region.

## DISCUSSION

Global data on childhood blindness as well as data from a study conducted in Canada suggest that in developing countries, preventable causes of childhood blindness were mainly due to corneal opacification caused by a combination of measles, xerophthalmia and the use of traditional eye medicine.^1^,^2^

Traditional medicine has been the only method of treatment available to the people living in the Sultanate of Oman until the 1980s. The country has shown remarkable development in the delivery of health care recently. Nevertheless, people still flock to traditional healers for relief from minor and major ailments hoping for a permanent cure to their ailments.^1^–^3^ A study on keratitis conducted at the tertiary care teaching institute in the Sultanate revealed that over 62.3% with infectious keratitis had sought traditional methods before presenting to the ophthalmologist.^4^ Al Shafee *et al.*, in their study on 400 patients with risk factors for stroke reported that 19.5% of subjects when questioned preferred to be treated at home first with indigenous treatments, 7.5% preferred to be treated exclusively at home with indigenous treatments, which included complete isolation or ‘Kanan’ of the patient along with ‘Wasam’, or branding, on certain points on the patient's body, and treatments utilizing herbs and herbal oil massage.^5^ In this study, we describe different mechanisms of serious ocular injury following application of traditional medications.

Traditional medicine, in Oman is based on the ancient Hippocratic-Greek method, the practice of which varies in different regions within the country. However, in general, herbal, spiritual and mechanical methods are applied as part of treatment.^1^–^4^ The common belief is that anything herbal and traditional implied absence of any risk, has lead to the development of herbal medicine as the most frequently used and lucrative form of traditional medicine.^1^,^3^ Herbal remedies are widely used for the treatment and prevention of various diseases and often contain highly active pharmacological compounds. Many medicinal herbs and pharmaceutical drugs are therapeutic at a particular dose and toxic or lethal at another dose. As traditional medicines become popular worldwide, toxicity related to these remedies also becomes more recognized.^1^–^3^ Patient 1 is an example of toxicity to the cornea following application of extract from the herb, *C. procera.*

*Calotropis procera*, a xerophytic shrub of family *Asclepiadaceae*, is widely used for treating various diseases, in the indigenous systems of medicine in India and other Asian and Middle Eastern countries.^6^ The milky white endogenous latex, produced by the plant exhibits a variety of effects in various animal models. On oral administration, the latex has potent anti-inflammatory, analgesic and weak antipyretic effects, while on local administration it induces an intense inflammatory response.^6^,^7^ These antagonistic biological activities (inflammatory and anti-inflammatory) depend on the extraction medium and route of administration in experimental animals.^6^

*Calotropis*-induced ocular injury can be from mechanical trauma or more commonly toxic, due to accidental exposure to the latex of the plant. Keratitis and iridocyclitis following accidental exposure to the plant juice has been reported previously.^6^,^7^ The keratitis is characterized by a relative lack of pain, corneal striae on the posterior corneal surface with corneal edema resulting in reduction in visual acuity. Corneal epithelial injury is rare and, if present, results from rubbing of the eyes.^6^ Our patient applied the leaf extract of the plant to treat the chalazion involving the left lower lid and developed decreased vision and corneal edema secondary to latex-related endothelial toxicity, which resolved with topical steroid therapy.

In ancient times, honey from Attica had a special reputation as a curative substance for eye disorders. Aristotle wrote in 350 BC in section 627a 3 of *Historia Animalium* that ‘White honey is good as a salve for sore eyes’. In other Asian countries, honey was used as a treatment for eye diseases, varying from blepharitis, conjunctivitis and infective keratitis, thermal and chemical burns to the eye.^8^–^11^

The role of honey as a remedy for the treatment of infected wounds, is being ‘rediscovered’ by the medical profession, particularly where conventional modern therapeutic agents have failed.^9^–^11^ Recent published reports describe the effectiveness of honey in rapidly clearing wound infection with minimal adverse effects, and also possible in promoting healing with minimal scar formation. Honey also has antimicrobial action against a broad spectrum of bacteria and fungi, both in laboratory studies and in humans.^10^–^12^ Its use in the eye ranges from treating post-herpetic corneal opacities, local conjunctival lesions and corneal edema with variable results.^10^,^13^ However, further research is needed to optimize the effective use of this agent in clinical ophthalmic practice.^8^–^13^ Patient 2, developed a hypopyon corneal ulcer following injury with a horse tail. Honey was applied to alleviate ocular pain. Isolation of dermatophyte *T. mentagrophyte*, from the ulcer, in the absence of any fungal skin, hair or nail disease, indicates the animal as the probable source for the infection. A similar case of keratitis due to *T. mentagrophyte,* following animal tail injury and application of turmeric powder has been reported by the authors.^14^ Honey used by our patient alleviated her ocular pain, but did not cure the fungal keratitis.

‘*Wasam*’ or ‘*kaii*’ is the colloquial term used to describe a mechanical method of healing by cauterization. It is a crude method of applying a counter irritant that is considered as a method to treat diseases such as ‘leg swelling’, hepatitis, malignancies, aural and thyroid pain.^15^,^16^ It has been widely used by faith healers in Egypt and by pre-Islamic Arabs centuries ago, and is still used in Oman as a last resort when modern medicine fails to cure illness.^15^–^17^ The instrument for cauterization is usually a metal rod that is pointed at one end or bent at the top into a crescent shape. Different parts of the body are cauterized for different ailments and diseases. For eye diseases, the back of the ear on the ipsilateral side is cauterized initially. If no relief, or effect is seen, the opposite side is treated.^15^–^18^ The wounds are then treated with herbal medicine. Abou-Elhamd reported two patients who underwent wasam treatment for aural pain and thyroid pain, respectively. The latter was found to have thyroid malignancy later.^18^ Wasam treatment has resulted in severe medical complications such as septic shock, tetanus, multiple splenic abscesses and cavernous sinus thrombosis.^15^–^18^

In our patient, wasam was applied on the occipital area rather than behind the ears, where it is typically applied for ocular diseases. That might have been done because the occiput was easily accessible for application in a child. Though there were no serious systemic complications, the child had to be hospitalized for a month until the ocular inflammation and secondary glaucoma were controlled and occipital lesions healed. Wasam application, resulted in scars on the occiput, but did not have a direct causal effect to the ocular inflammation or elevated intraocular pressure noted in this patient. It is likely that the discontinuation of the medications prescribed during the postoperative period triggered intraocular inflammation causing secondary glaucoma and poor postoperative visual recovery.

Traditional healing practices reflect the culture and beliefs of a society.^19^,^20^ Azaizeh *et al.*, in their review on the re-emergence of Traditional Arabic and Islamic medicine believed that, the remarkable success of traditional medicine in treating many acute and chronic ailments was the reason behind it being chosen by many in the Mediterranean region, as the first choice for treatment of infertility, epilepsy, psychosomatic troubles and depression, despite lack of scientific data supporting its efficacy or safety.^1^–^20^ Oman has one of the world's best healthcare delivery system providing free healthcare services to the natives. Nevertheless, people of Oman seek help from traditional healers. The deep faith, belief and trust that the Omani people have in the traditional healers and their ignorance to the harm caused by injudicious usage of this form of medicine and practice is highlighted in this report.
